# Supplementary figures and images for: E-cadherin bridges cell polarity and spindle orientation to ensure prostate epithelial integrity and prevent carcinogenesis in vivo
Source: PLoS Genet. 2018 Aug 17;14(8):e1007609. doi: 10.1371/journal.pgen.1007609 (PMC6115016; doi:10.1371/journal.pgen.1007609)

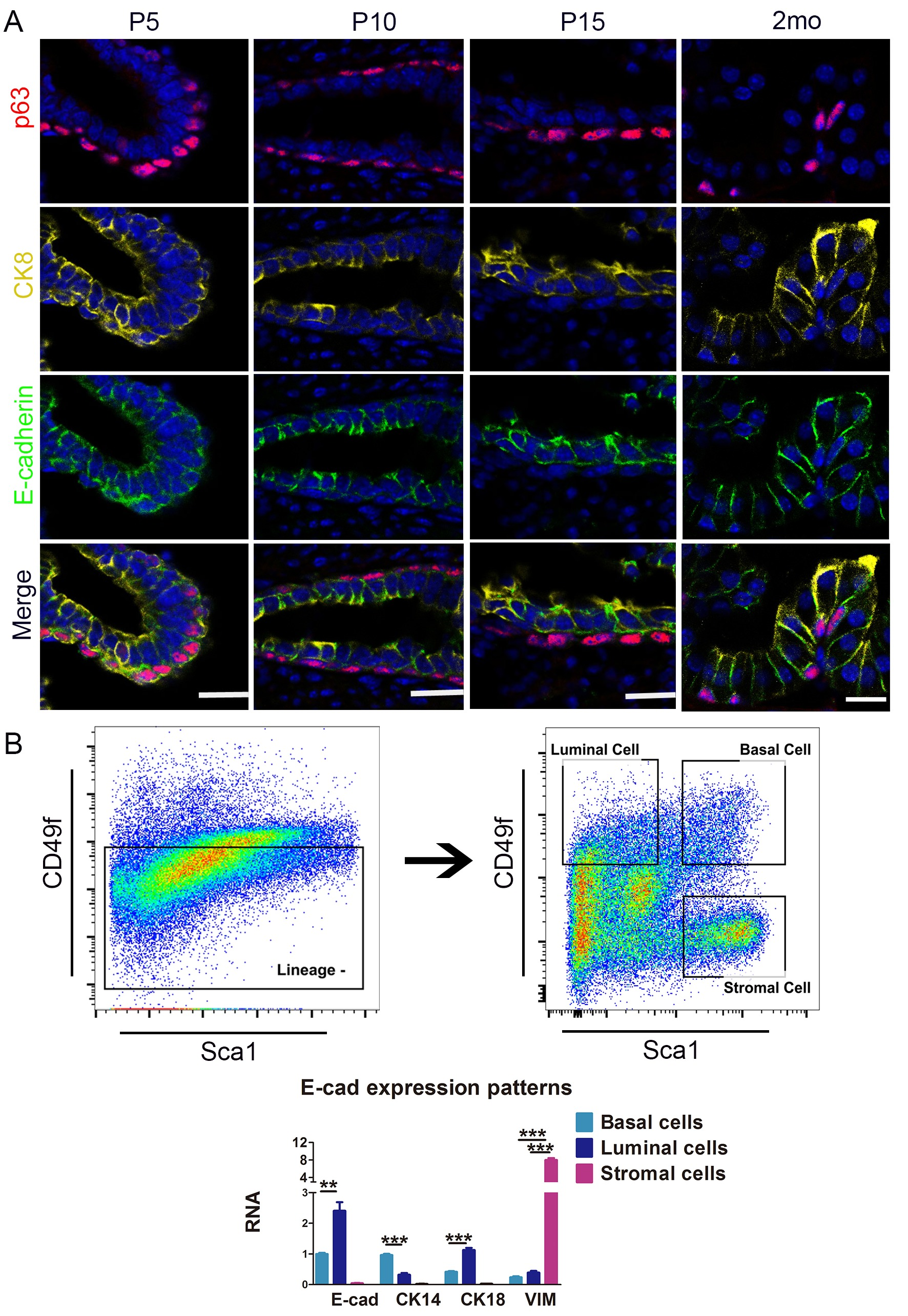

Supplement: S1 Fig — (A) Confocal images showing expression patterns of E-cadherin at indicated developmental stages. Scale bars are 20μm. (B) RT-PCR analysis shows that E-cadherin is predominantly expressed in prostate luminal cells. Luminal, basal and stromal cells from 2-month old mice were enriched using flow cytometric sorting based on lineage, Sca1 and CD49f staining. (n = 3. Data are presented as mean ± s.e.m, and the P value was determined by the Student’s t-test, ***P<0.01, **P<0.05) (TIF) [file pgen.1007609.s001.tif]

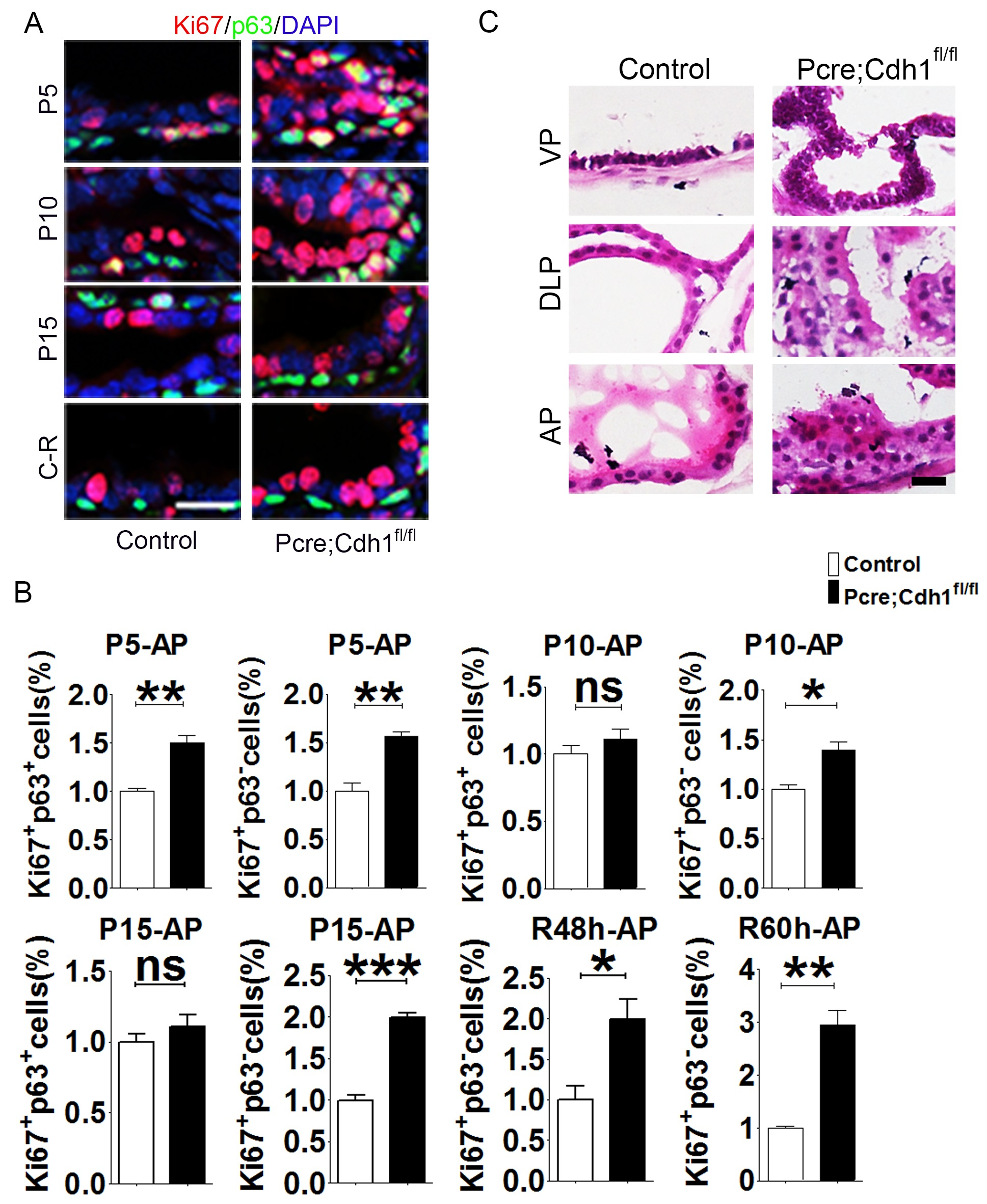

Supplement: S2 Fig — (A) Sections of anterior lobes (AP) of developing or regenerating prostates at indicated time points were stained with the proliferative marker Ki67 and basal cells marker p63. Slides were counterstained by DAPI. (n = 3) (B) Quantification of Ki67 positive cells suggests that E-cadherin knockout leads to hyperproliferation of luminal cells in prostate development and regeneration. (Student’s t-test, **P<0.01, *P<0.05, error bars = SEM. n = 3) (C) H&E staining shows that multilayered epithelia structure can be early found in 6-week-old E-cadherin knockout mouse prostates. (Scale bars are 20μm.for (A) and (C), n = 3) (TIF) [file pgen.1007609.s002.tif]

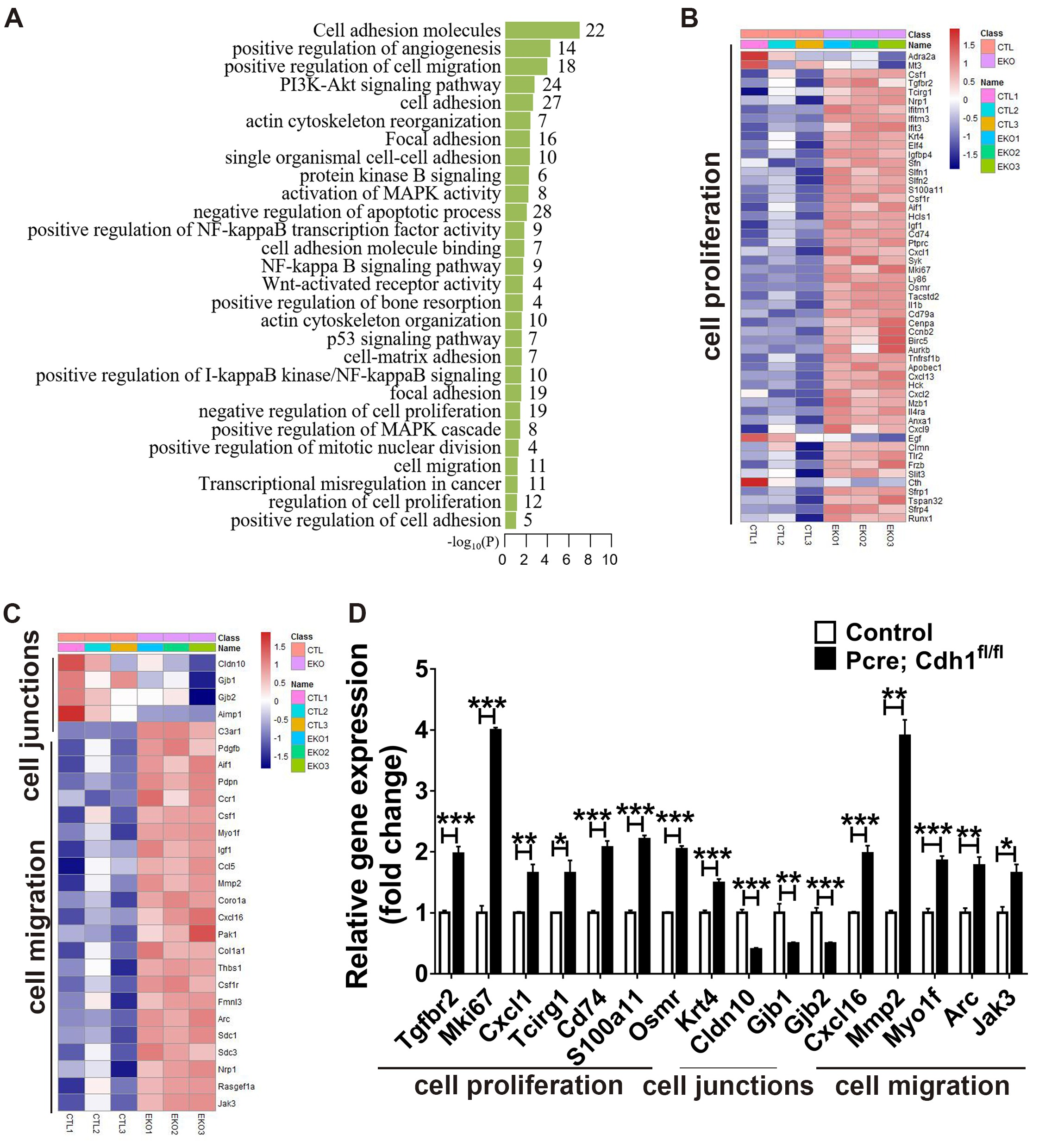

Supplement: S3 Fig — (A) Gene Ontology (GO) analysis reveals an enrichment of cell proliferation, migration and cell junctions associated molecules or signaling pathways. (B) A heatmap showing the expression levels for cell proliferation associated genes. (C) A heatmap illustrating the expression levels for cell junctions and cell migration associated genes. (D) qRT-PCR data confirm hyperproliferative and malignant phenotypes in transgenic mice. (TIF) [file pgen.1007609.s003.tif]

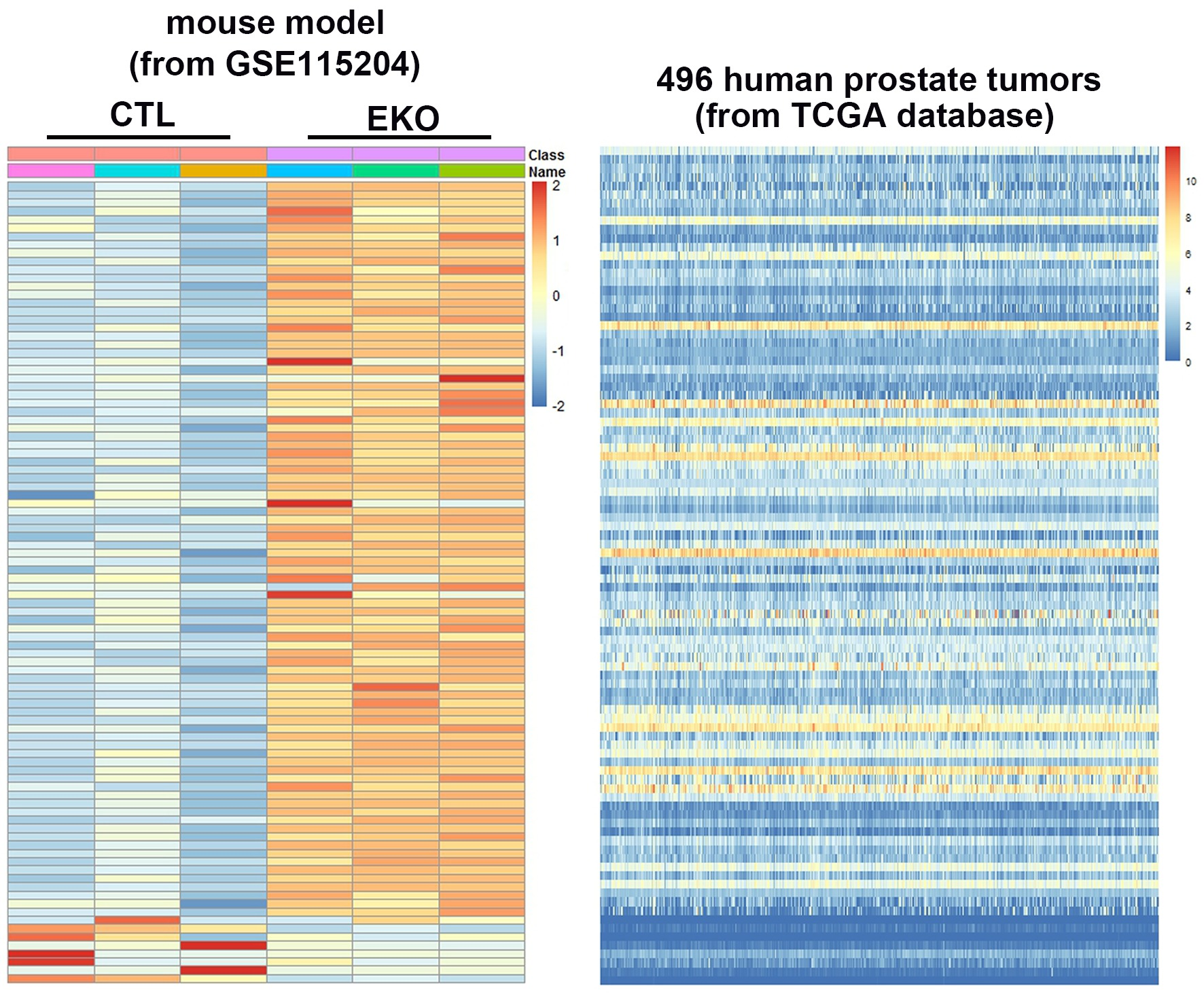

Supplement: S4 Fig — Patterns of E-cadherin-regulated signature genes which were represented in the TCGA database of human prostate cancer expression profiles. (TIF) [file pgen.1007609.s004.tif]

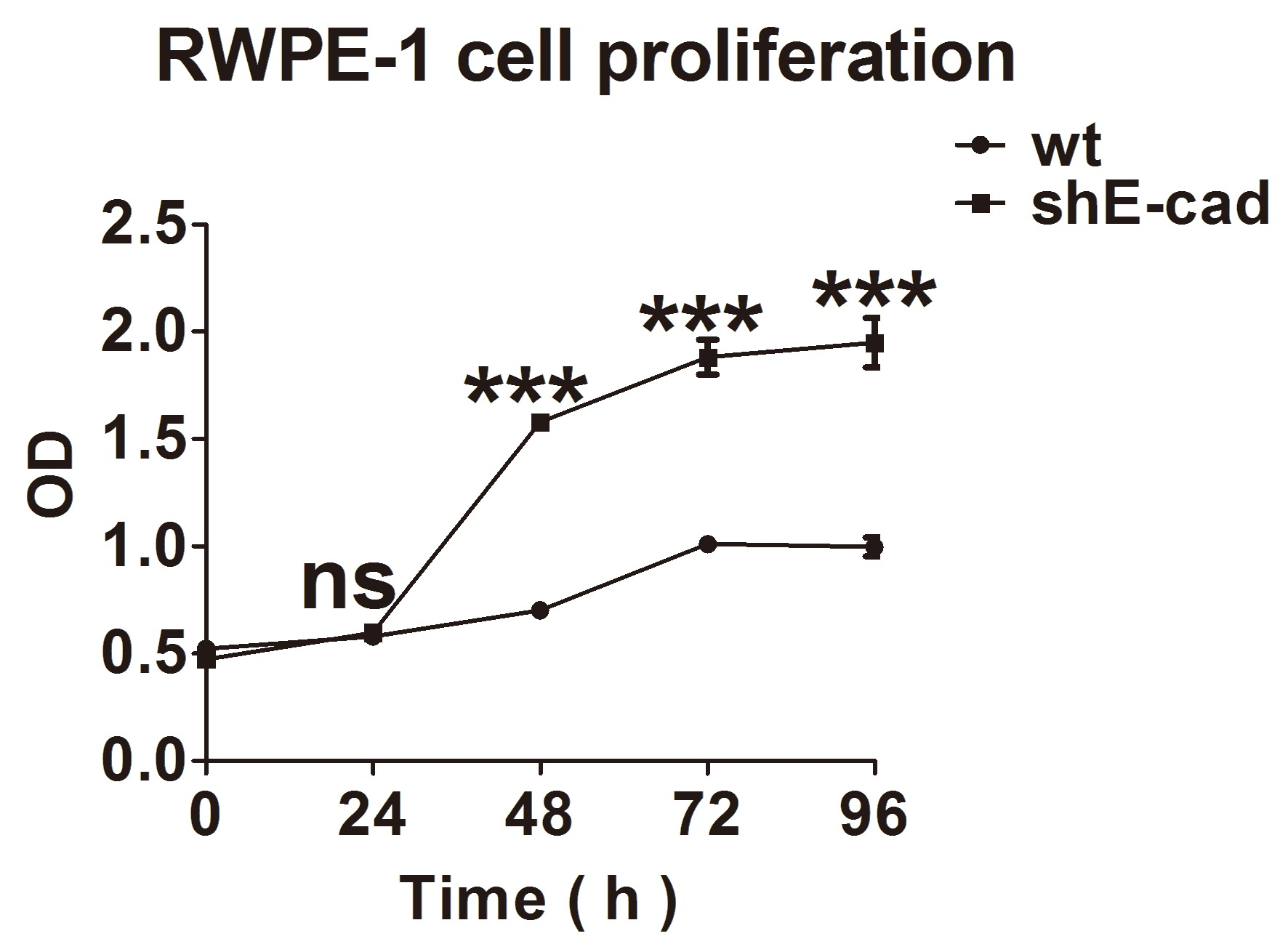

Supplement: S5 Fig — Cell number was determined by a Cell Counting Kit 8 assay (n = 5 duplicates). (TIF) [file pgen.1007609.s005.tif]

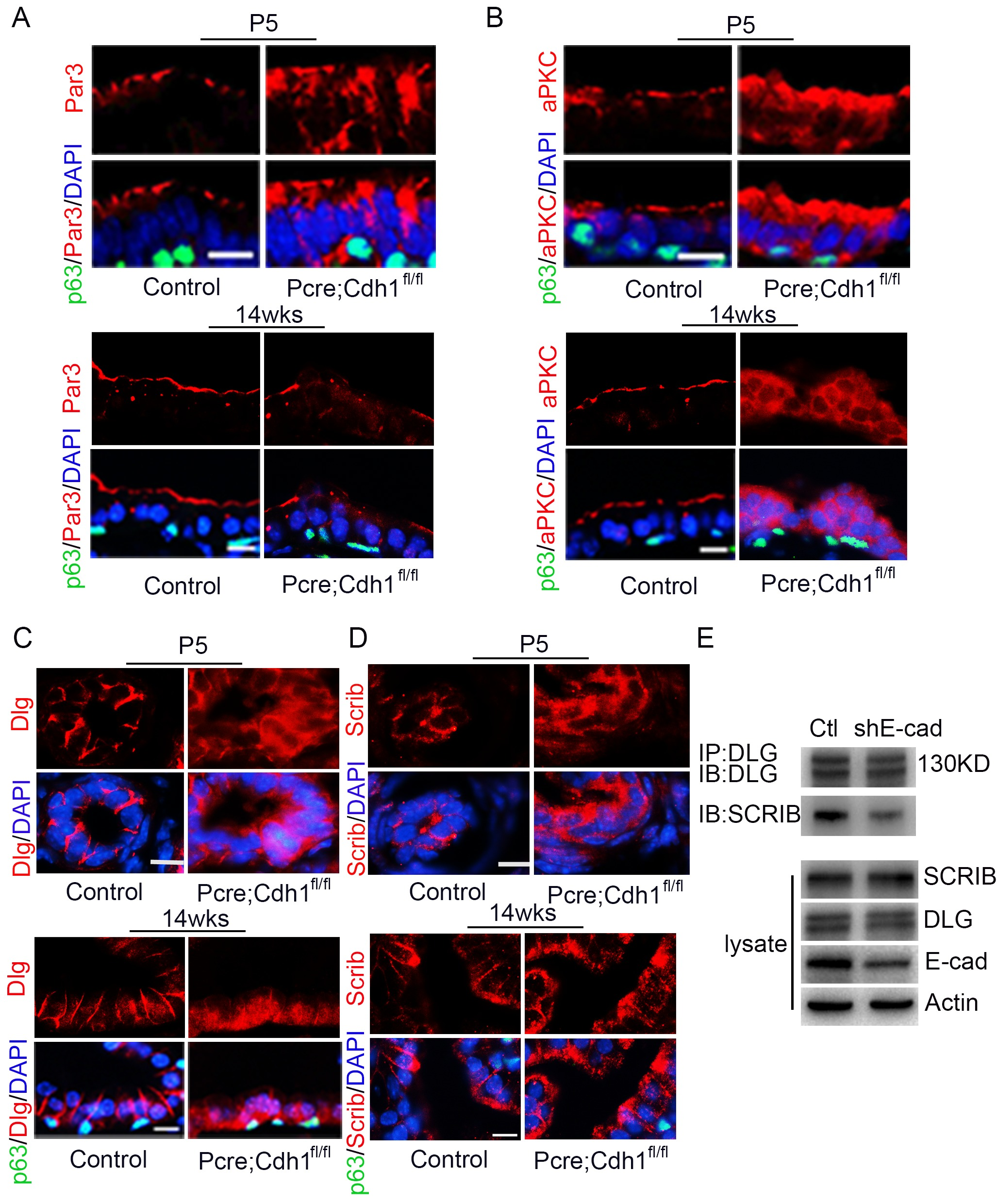

Supplement: S6 Fig — (A-B) Sections of P5 and adult prostates were stained with antibodies against PAR3 (A), aPKC (B) and p63. Apical distribution of PAR3 or aPKC in luminal cells were disrupted by E-cadherin knockout. (n = 3) (C-D) Immunostaining of P5 and adult prostates shows that normal basolateral localization of polarity proteins DLG-1 (C) and SCRIB (D) become diffused in E-cadherin deleted luminal cells. (All sections are counterstained with DAPI. Scale bars are 10μm.n = 3) (E)The formation of DLG/SCRIB protein complex is markedly suppressed due to E-cadherin knockdown in RWPE-1 cells. (TIF) [file pgen.1007609.s006.tif]

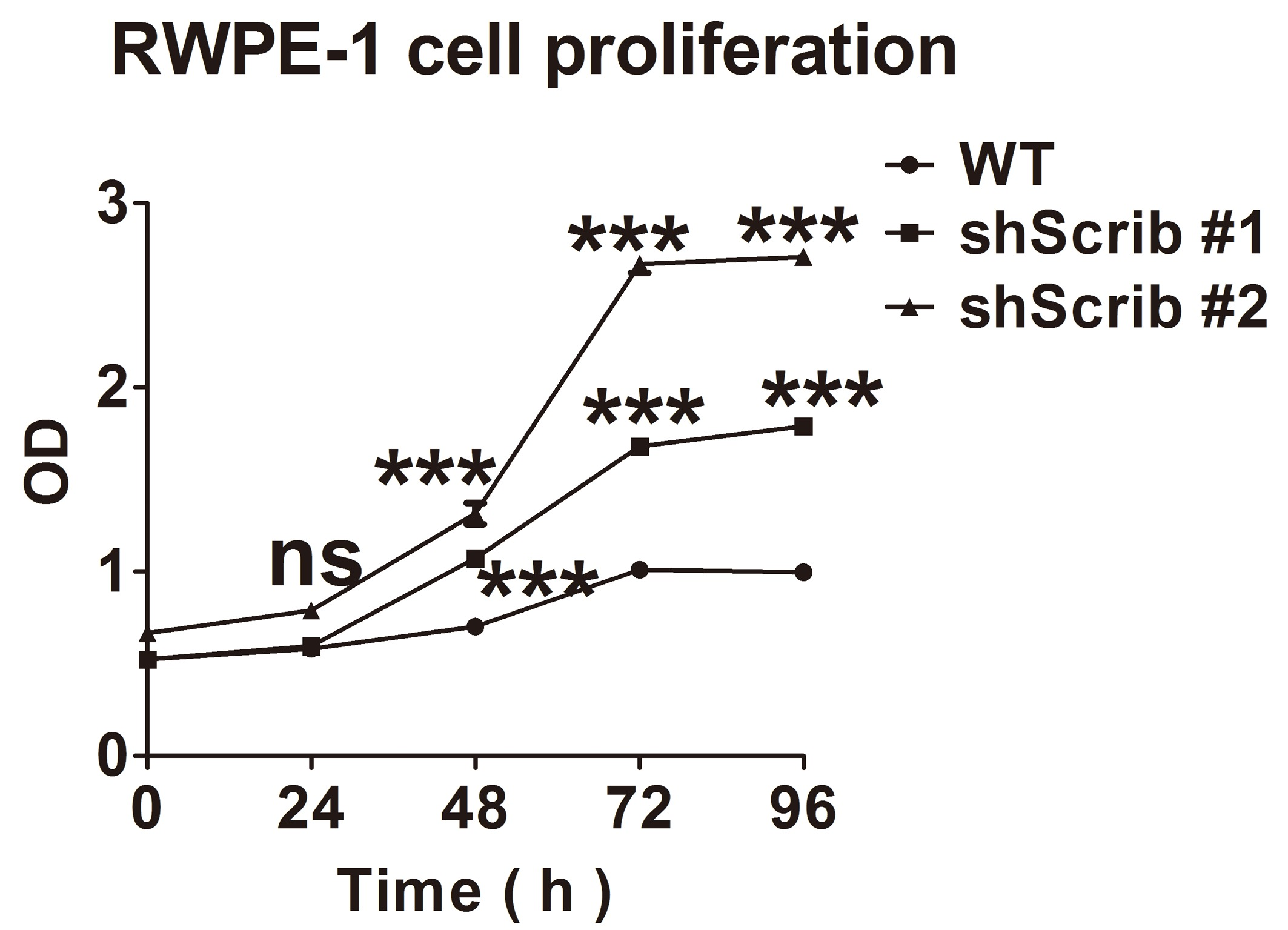

Supplement: S7 Fig — Cell number was determined by a Cell Counting Kit 8 assay (n = 5 duplicates). (TIF) [file pgen.1007609.s007.tif]
