# Supplementary material for: E-cadherin bridges cell polarity and spindle orientation to ensure prostate epithelial integrity and prevent carcinogenesis in vivo
Source: PLoS Genet. 2018 Aug 17;14(8):e1007609. doi: 10.1371/journal.pgen.1007609 (PMC6115016; doi:10.1371/journal.pgen.1007609)
Supplement: S1 Table — (DOCX) [file pgen.1007609.s008.docx]

**S1 Table. Quantification of percentage of proliferative epithelial cells in different prostate lobes during postnatal development and regeneration**

|  | P5-AP | | | | | |
| --- | --- | --- | --- | --- | --- | --- |
| genotype | Control | | | *Pcre;Cdh1^fl/fl^* | | |
| mouse number | #1 | #2 | #3 | #1 | #2 | #3 |
| Basal cells | 945 | 917 | 812 | 1239 | 1029 | 925 |
| Luminal cells | 1235 | 1120 | 1041 | 1527 | 1215 | 1089 |
| Ki67^+^ basal cells | 204 | 187 | 159 | 389 | 289 | 306 |
| Ki67^+^ luminal cells | 496 | 475 | 431 | 814 | 598 | 611 |
| Ki67^+^ basal cells (percentage) | 22% | 20% | 19% | 31% | 28% | 33% |
| Ki67^+^ luminal cells (percentage) | 40% | 42% | 41% | 53% | 49% | 56% |
| Basal cells (percentage) | 43% | 45% | 44% | 45% | 46% | 46% |
| Luminal cells (percentage) | 57% | 55% | 56% | 55% | 54% | 54% |
|  | P5-VP/DLP | | | | | |
| genotype | Control | | | *Pcre;Cdh1^fl/fl^* | | |
| mouse number | #1 | #2 | #3 | #1 | #2 | #3 |
| Basal cells | 451 | 329 | 395 | 417 | 527 | 428 |
| Luminal cells | 627 | 472 | 543 | 561 | 691 | 591 |
| Ki67^+^ basal cells | 87 | 83 | 93 | 153 | 183 | 164 |
| Ki67^+^ luminal cells | 295 | 247 | 235 | 342 | 379 | 330 |
| Ki67^+^ basal cells (percentage) | 19% | 25% | 24% | 37% | 35% | 38% |
| Ki67^+^ luminal cells (percentage) | 47% | 52% | 43% | 61% | 55% | 56% |
| Basal cells (percentage) | 42% | 41% | 42% | 43% | 43% | 42% |
| Luminal cells (percentage) | 58% | 59% | 58% | 57% | 57% | 58% |

|  | P10-AP | | | | | |
| --- | --- | --- | --- | --- | --- | --- |
| genotype | Control | | | *Pcre;Cdh1^fl/fl^* | | |
| mouse number | #1 | #2 | #3 | #1 | #2 | #3 |
| Basal cells | 741 | 503 | 623 | 638 | 1121 | 572 |
| Luminal cells | 1236 | 925 | 1002 | 1202 | 2071 | 1129 |
| Ki67^+^ basal cells | 132 | 98 | 96 | 110 | 218 | 125 |
| Ki67^+^ luminal cells | 259 | 203 | 241 | 415 | 576 | 350 |
| Ki67^+^ basal cells (percentage) | 19% | 20% | 15% | 17% | 20% | 22% |
| Ki67^+^ luminal cells (percentage) | 21% | 22% | 24% | 35% | 28% | 31% |
| Basal cells (percentage) | 38% | 35% | 38% | 35% | 35% | 33% |
| Luminal cells (percentage) | 62% | 65% | 62% | 65% | 65% | 67% |
|  | P10-VP/DLP | | | | | |
| genotype | Control | | | *Pcre;Cdh1^fl/fl^* | | |
| mouse number | #1 | #2 | #3 | #1 | #2 | #3 |
| Basal cells | 531 | 581 | 557 | 549 | 562 | 572 |
| Luminal cells | 1239 | 1494 | 1193 | 1561 | 1519 | 1619 |
| Ki67^+^ basal cells | 91 | 127 | 116 | 123 | 110 | 138 |
| Ki67^+^ luminal cells | 326 | 478 | 341 | 688 | 633 | 779 |
| Ki67^+^ basal cells (percentage) | 17% | 22% | 21% | 22% | 20% | 24% |
| Ki67^+^ luminal cells (percentage) | 26% | 32% | 29% | 44% | 42% | 48% |
| Basal cells (percentage) | 30% | 28% | 32% | 26% | 27% | 26% |
| Luminal cells (percentage) | 70% | 72% | 68% | 74% | 73% | 74% |

|  | | P15-AP | | | | | |
| --- | --- | --- | --- | --- | --- | --- | --- |
| genotype | | Control | | | *Pcre;Cdh1^fl/fl^* | | |
| mouse number | | #1 | #2 | #3 | #1 | #2 | #3 |
| Basal cells | | 950 | 632 | 812 | 762 | 848 | 673 |
| Luminal cells | | 1815 | 1168 | 1628 | 2609 | 2544 | 2109 |
| Ki67^+^ basal cells | | 148 | 121 | 139 | 142 | 142 | 147 |
| Ki67^+^ luminal cells | | 537 | 277 | 425 | 1359 | 1409 | 1065 |
| Ki67^+^ basal cells (percentage) | | 16% | 19% | 17% | 19% | 17% | 22% |
| Ki67^+^ luminal cells (percentage) | | 30% | 24% | 26% | 52% | 55% | 51% |
| Basal cells (percentage) | | 34% | 35% | 33% | 23% | 25% | 24% |
| Luminal cells (percentage) | | 66% | 65% | 67% | 77% | 75% | 76% |
|  | | P15-VP/DLP | | | | | |
| genotype | | Control | | | *Pcre;Cdh1^fl/fl^* | | |
| mouse number | | #1 | #2 | #3 | #1 | #2 | #3 |
| Basal cells | | 548 | 557 | 574 | 582 | 567 | 563 |
| Luminal cells | | 1164 | 1086 | 1277 | 2465 | 2226 | 2105 |
| Ki67^+^ basal cells | | 115 | 130 | 104 | 112 | 124 | 136 |
| Ki67^+^ luminal cells | | 371 | 383 | 346 | 1302 | 1249 | 1240 |
| Ki67^+^ basal cells (percentage) | | 21% | 23% | 18% | 19% | 22% | 24% |
| Ki67^+^ luminal cells (percentage) | | 32% | 35% | 27% | 59% | 56% | 59% |
| Basal cells (percentage) | | 32% | 34% | 31% | 19% | 20% | 21% |
| Luminal cells (percentage) | | 68% | 66% | 69% | 81% | 80% | 79% |
|  | R48h（AP） | | | | | | |
| genotype | Control | | | | *Pcre;Cdh1^fl/fl^* | | |
| mouse number | #1 | | #2 | #3 | #1 | #2 | #3 |
| Luminal cells | 290 | | 216 | 360 | 240 | 225 | 250 |
| Ki67^+^ luminal cells | 75 | | 90 | 90 | 180 | 110 | 153 |
| Ki67^+^ luminal cells (percentage) | 26% | | 42% | 25% | 75% | 49% | 61% |
|  | | R48h（VP/DLP） | | | | | |
| genotype | | Control | | | *Pcre;Cdh1^fl/fl^* | | |
| mouse number | | #1 | #2 | #3 | #1 | #2 | #3 |
| Luminal cells | | 110 | 267 | 159 | 124 | 165 | 138 |
| Ki67^+^ luminal cells | | 20 | 61 | 41 | 50 | 63 | 69 |
| Ki67^+^ luminal cells (percentage) | | 18% | 23% | 26% | 40% | 38% | 50% |
|  | | R60h（AP） | | | | | |
| genotype | | Control | | | *Pcre;Cdh1^fl/fl^* | | |
| mouse number | | #1 | #2 | #3 | #1 | #2 | #3 |
| Luminal cells | | 251 | 214 | 297 | 235 | 219 | 284 |
| Ki67^+^ luminal cells | | 55 | 51 | 62 | 182 | 124 | 176 |
| Ki67^+^ luminal cells (percentage) | | 22% | 24% | 21% | 77% | 57% | 62% |
|  | | R60h（VP/DLP） | | | | | |
| genotype | | Control | | | *Pcre;Cdh1^fl/fl^* | | |
| mouse number | | #1 | #2 | #3 | #1 | #2 | #3 |
| Luminal cells | | 124 | 108 | 135 | 128 | 117 | 141 |
| Ki67^+^ luminal cells | | 19 | 21 | 29 | 53 | 55 | 73 |
| Ki67^+^ luminal cells (percentage) | | 15% | 19% | 22% | 41% | 47% | 52% |
