# Supplementary material for: E-cadherin bridges cell polarity and spindle orientation to ensure prostate epithelial integrity and prevent carcinogenesis in vivo
Source: PLoS Genet. 2018 Aug 17;14(8):e1007609. doi: 10.1371/journal.pgen.1007609 (PMC6115016; doi:10.1371/journal.pgen.1007609)
Supplement: S2 Table — (DOCX) [file pgen.1007609.s009.docx]

**S2 Table. Quantification of percentages of normal, PIN and adenocarcinoma phenotypes of the prostatic histology in 9-mo and 21-mo old Ecadherin knockout mice.**

| 9-mo old *Pcre; Cdh1^fl/fl^* mice | | | | |
| --- | --- | --- | --- | --- |
| Mouse | Normal  (Lumen num./per.) | PIN  (Lumen num./per.) | Adenocarcinoma  (Lumen num./per.) | Total |
| #1 | 18/42.9% | 24/57.1% | 0 | 42 |
| #2 | 29/59.2% | 20/40.8% | 0 | 49 |
| #3 | 10/10.8% | 83/89.2% | 0 | 93 |
| #4 | 21/36.8% | 36/63.2% | 0 | 57 |
| #5 | 23/26.7% | 63/73.3% | 0 | 86 |
| #6 | 10/23.8% | 32/76.2% | 0 | 42 |
| Total | 111 | 258 | 0 | 369 |

| 21-mo old *Pcre; Cdh1^fl/fl^* mice | | | | |
| --- | --- | --- | --- | --- |
| Mouse | Normal  (Lumen num./per.) | PIN  (Lumen num./per.) | Adenocarcinoma  (Lumen num./per.) | Total |
| #1 | 10/14.3% | 60/85.7% | 0 | 70 |
| #2 | 2/4.2% | 44/91.6% | 2/4.2% | 48 |
| #3 | 10/18.2% | 45/81.8% | 0 | 55 |
| #4 | 8/10.1% | 68/86.1% | 3/3.8% | 79 |
| #5 | 9/10.0% | 81/90.0% | 0 | 90 |
| #6 | 9/20.5% | 35/79.5% | 0 | 44 |
| #7 | 10/23.3% | 33/76.7% | 0 | 43 |
| #8 | 7/9.2% | 67/88.2% | 2/2.6% | 76 |
| Total | 65 | 433 | 7 | 505 |
