# Supplementary material for: E-cadherin bridges cell polarity and spindle orientation to ensure prostate epithelial integrity and prevent carcinogenesis in vivo
Source: PLoS Genet. 2018 Aug 17;14(8):e1007609. doi: 10.1371/journal.pgen.1007609 (PMC6115016; doi:10.1371/journal.pgen.1007609)
Supplement: S4 Table — (DOCX) [file pgen.1007609.s011.docx]

**S4 Table. Quantification of percentages of horizontal, vertical and titled divisions in mitotic epithelial cells at different murine prostate development and regeneration stages, as well as in RWPE-1 cells of different cell cycle phases.**

| P5 of control mice | | | | |
| --- | --- | --- | --- | --- |
| Mouse | Horizontal  (num./per.) | Vertical  (num./per.) | Tilted  (num./per.) | Total |
| #1 | 28/87.5% | 3/9.4% | 1/3.1% | 32 |
| #2 | 23/82.1% | 3/10.7% | 2/7.1% | 28 |
| #3 | 36/87.8% | 3/7.3% | 2/4.9% | 41 |
| Total | 86 | 10 | 5 | 101 |

| P5 of *Pcre; Cdh1^fl/fl^* mice | | | | |
| --- | --- | --- | --- | --- |
| Mouse | Horizontal  (num./per.) | Vertical  (num./per.) | Tilted  (num./per.) | Total |
| #1 | 14/60.9% | 4/17.4% | 5/21.7% | 23 |
| #2 | 16/57.1% | 5/17.9% | 7/25.0% | 28 |
| #3 | 13/61.9% | 3/14.3% | 5/23.8% | 21 |
| Total | 43 | 12 | 17 | 72 |

| P10 of control mice | | | | |
| --- | --- | --- | --- | --- |
| Mouse | Horizontal  (num./per.) | Vertical  (num./per.) | Tilted  (num./per.) | Total |
| #1 | 26/81.3% | 4/12.5% | 2/6.3% | 32 |
| #2 | 31/86.1% | 3/8.3% | 2/5.6% | 36 |
| #3 | 33/82.5% | 4/10.0% | 3/7.5% | 40 |
| Total | 90 | 11 | 7 | 108 |

| P10 of *Pcre; Cdh1^fl/fl^* mice | | | | |
| --- | --- | --- | --- | --- |
| Mouse | Horizontal  (num./per.) | Vertical  (num./per.) | Tilted  (num./per.) | Total |
| #1 | 13/52.0% | 7/28.0% | 5/20.0% | 25 |
| #2 | 15/55.6%% | 6/22.2% | 6/22.2% | 27 |
| #3 | 11/57.9% | 5/26.3% | 3/15.8% | 19 |
| Total | 47 | 14 | 10 | 71 |

| P15 of control mice | | | | |
| --- | --- | --- | --- | --- |
| Mouse | Horizontal  (num./per.) | Vertical  (num./per.) | Tilted  (num./per.) | Total |
| #1 | 19/82.6% | 1/4.3% | 3/13.0% | 23 |
| #2 | 29/82.9% | 2/5.7% | 4/11.4% | 35 |
| #3 | 37/86.0% | 2/4.7% | 4/9.3% | 43 |
| Total | 85 | 5 | 11 | 101 |

| P15 of *Pcre; Cdh1^fl/fl^* mice | | | | |
| --- | --- | --- | --- | --- |
| Mouse | Horizontal  (num./per.) | Vertical  (num./per.) | Tilted  (num./per.) | Total |
| #1 | 18/58.1% | 6/19.4% | 7/22.5% | 31 |
| #2 | 24/60.0% | 6/15.0% | 10/25.0% | 40 |
| #3 | 25/55.6% | 8/17.8% | 12/26.6% | 45 |
| Total | 85 | 16 | 15 | 116 |

| R48h of control mice | | | | |
| --- | --- | --- | --- | --- |
| Mouse | Horizontal | Vertical | Tilted | Total |
| #1 | 23/79.3% | 1/3.4% | 5/17.2% | 29 |
| #2 | 27/81.8% | 1/3.0% | 5/15.2% | 33 |
| #3 | 28/80.0% | 2/5.7% | 5/14.3% | 35 |
| Total | 78 | 4 | 15 | 97 |

| R48h of *Pcre; Cdh1^fl/fl^* mice | | | | |
| --- | --- | --- | --- | --- |
| Mouse | Horizontal | Vertical | Tilted | Total |
| #1 | 15/55.6% | 3/11.1% | 9/33.3% | 27 |
| #2 | 14/58.3% | 2/8.3% | 8/33.4% | 24 |
| #3 | 16/53.3% | 3/10.0% | 11/36.7% | 30 |
| Total | 53 | 4 | 24 | 81 |

| R60h of control mice | | | | |
| --- | --- | --- | --- | --- |
| Mouse | Horizontal | Vertical | Tilted | Total |
| #1 | 24/77.4% | 2/6.5% | 5/16.1% | 31 |
| #2 | 19/73.1% | 1/3.8% | 6/23.1% | 26 |
| #3 | 23/76.7% | 2/6.7% | 5/16.7% | 30 |
| Total | 66 | 5 | 16 | 87 |

| R60h of *Pcre; Cdh1^fl/fl^* mice | | | | |
| --- | --- | --- | --- | --- |
| Mouse | Horizontal | Vertical | Tilted | Total |
| #1 | 13/50.0% | 2/7.7% | 11/42.3% | 26 |
| #2 | 17/53.1% | 2/5.6% | 13/40.6% | 32 |
| #3 | 17/51.5% | 3/9.1% | 13/39.4% | 33 |
| Total | 47 | 7 | 37 | 91 |

| Metaphase of RWPE-1 cells | | | | |
| --- | --- | --- | --- | --- |
| Control | Horizontal | Vertical | Tilted | Total |
| 1st | 17/65.4% | 2/7.7% | 7/26.9% | 26 |
| 2nd | 15/62.5% | 1/4.2% | 8/33.3% | 24 |
| 3rd | 18/60.0% | 2/6.7% | 10/33.3% | 30 |
| **Total** | 50 | 5 | 25 | 80 |
| shCdh1 | Horizontal | Vertical | Tilted | Total |
| 1st | 14/48.3% | 4/13.8% | 11/37.9% | 29 |
| 2nd | 14/53.8% | 3/11.5% | 9/34.6% | 26 |
| 3rd | 18/47.4% | 5/13.2% | 15/39.5% | 38 |
| **Total** | 46 | 12 | 35 | 93 |

| \| Telophase of RWPE-1 cells \| \| \| \| \| \| --- \| --- \| --- \| --- \| --- \| \| Control \| Horizontal \| Vertical \| Tilted \| Total \| \| 1st \| 34/82.9% \| 1/2.4% \| 6/14.6% \| 41 \| \| 2nd \| 42/84.0% \| 2/4.0% \| 6/12.0% \| 50 \| \| 3rd \| 45/83.3% \| 2/3.7% \| 7/13.0% \| 54 \| \| **Total** \| 121 \| 5 \| 19 \| 145 \| \| shCdh1 \| Horizontal \| Vertical \| Tilted \| Total \| \| 1st \| 40/56.3% \| 9/12.7% \| 22/31.0% \| 71 \| \| 2nd \| 29/51.8% \| 8/14.3% \| 19/33.9% \| 56 \| \| 3rd \| 29/58.0% \| 7/14.0% \| 14/28.0% \| 50 \| \| **Total** \| 122 \| 12 \| 43 \| 177 \| |
| --- | --- | --- | --- | --- | --- | --- | --- | --- | --- | --- | --- | --- | --- | --- | --- | --- | --- | --- | --- | --- | --- | --- | --- | --- | --- | --- | --- | --- | --- | --- | --- | --- | --- | --- | --- | --- | --- | --- | --- | --- | --- | --- | --- | --- | --- | --- | --- | --- | --- | --- | --- | --- | --- | --- | --- |
