# Supplementary material for: E-cadherin bridges cell polarity and spindle orientation to ensure prostate epithelial integrity and prevent carcinogenesis in vivo
Source: PLoS Genet. 2018 Aug 17;14(8):e1007609. doi: 10.1371/journal.pgen.1007609 (PMC6115016; doi:10.1371/journal.pgen.1007609)
Supplement: S5 Table — (DOCX) [file pgen.1007609.s012.docx]

**S5 Table. Quantification of percentages of each type of LGN and NUMA distributions in dividing luminal cells in different prostate development and regeneration stages**

| P5 of control mice (LGN) | | | | | |
| --- | --- | --- | --- | --- | --- |
|  | Cortical | A-B | A-O | B-O | Total |
|  | 36 | 3 | 1 | 1 | 41 |
| Percentage | 87.80% | 7.32% | 2.44% | 2.44% |  |

| P5 of *Pcre; Cdh1^fl/fl^* mice (LGN) | | | | | |
| --- | --- | --- | --- | --- | --- |
|  | Cortical | A-B | A-O | B-O | Total |
|  | 12 | 19 | 2 | 4 | 37 |
| Percentage | 32.43% | 51.35% | 5.41% | 10.81% |  |

| P15 of control mice (LGN) | | | | | |
| --- | --- | --- | --- | --- | --- |
|  | Cortical | A-B | A-O | B-O | Total |
|  | 36 | 3 | 3 | 1 | 43 |
| Percentage | 83.72% | 6.98% | 6.98% | 2.32% |  |

| P15 of *Pcre; Cdh1^fl/fl^* mice (LGN) | | | | | |
| --- | --- | --- | --- | --- | --- |
|  | Cortical | A-B | A-O | B-O | Total |
|  | 13 | 18 | 3 | 5 | 39 |
| Percentage | 33.33% | 46.15% | 7.69% | 12.83% |  |

| R48h of control mice (LGN) | | | | | |
| --- | --- | --- | --- | --- | --- |
|  | Cortical | A-B | A-O | B-O | Total |
|  | 33 | 5 | 1 | 3 | 42 |
| Percentage | 78.57% | 11.90% | 2.38% | 7.15% |  |

| R48h of *Pcre; Cdh1^fl/fl^* mice (LGN) | | | | | |
| --- | --- | --- | --- | --- | --- |
|  | Cortical | A-B | A-O | B-O | Total |
|  | 16 | 17 | 3 | 2 | 38 |
| Percentage | 42.11% | 44.74% | 7.89% | 5.26% |  |

| P5 of control mice (NUMA) | | | |
| --- | --- | --- | --- |
|  | Cortical | Smear | Total |
|  | 53 | 6 | 59 |
| Percentage | 89.83% | 10.17% |  |

| P5 of *Pcre; Cdh1^fl/fl^* mice (NUMA) | | | |
| --- | --- | --- | --- |
|  | Cortical | Smear | Total |
|  | 15 | 33 | 48 |
| Percentage | 31.25% | 68.75% |  |

| P15 of control mice (NUMA) | | | |
| --- | --- | --- | --- |
|  | Cortical | Smear | Total |
|  | 64 | 7 | 71 |
| Percentage | 90.14% | 9.86% |  |

| P15 of *Pcre; Cdh1^fl/fl^* mice (NUMA) | | | |
| --- | --- | --- | --- |
|  | Cortical | Smear | Total |
|  | 10 | 49 | 59 |
| Percentage | 16.95% | 83.05% |  |

| R48h of control mice (NUMA) | | | |
| --- | --- | --- | --- |
|  | Cortical | Smear | Total |
|  | 56 | 6 | 62 |
| Percentage | 90.32% | 9.68% |  |

| R48h of *Pcre; Cdh1^fl/fl^* mice (NUMA) | | | |
| --- | --- | --- | --- |
|  | Cortical | Smear | Total |
|  | 14 | 44 | 58 |
| Percentage | 24.14% | 75.86% |  |
