# Supplementary material for: E-cadherin bridges cell polarity and spindle orientation to ensure prostate epithelial integrity and prevent carcinogenesis in vivo
Source: PLoS Genet. 2018 Aug 17;14(8):e1007609. doi: 10.1371/journal.pgen.1007609 (PMC6115016; doi:10.1371/journal.pgen.1007609)
Supplement: S6 Table — (DOCX) [file pgen.1007609.s013.docx]

**S6 Table Antibodies used in this paper**

| Antibodies | | |
| --- | --- | --- |
| Anti-E-cadherin mouse monoclonal | BD | Cat#610181 |
| Anti-E-cadherin goat polyclonal | Abcam | Cat#ab128804 |
| Anti-p63 mouse monoclonal | Abcam | Cat#ab735 |
| Anti-Cytokeratin 5 rabbit monoclonal | Abcam | Cat#ab52635 |
| Anti-Cytokeratin 8 rabbit monoclonal | Abcam | Cat#ab53280 |
| Anti-Androgen Receptor rabbit monoclonal | Abcam | Cat#ab133273 |
| Anti-Ki67 rabbit polyclonal | Abcam | Cat#ab15580 |
| Anti-Survivin goat polyclonal | Santa Cruz | Cat#sc-8807 |
| Anti-gama-tubulin mouse monoclonal | Sigma | Cat#T6557 |
| Anti-ZO-1 mouse monoclonal | Life technologies | Cat#339100 |
| Anti-Partitioning defective 3 rabbit polyclonal | Upstate | Cat#07330 |
| Anti-aPKC rabbit polyclonal | Santa Cruz | Cat#sc-216 |
| Anti-SAP97 rabbit polyclonal | Santa Cruz | Cat#sc-25661 |
| Anti-Scrib goat polyclonal | Santa Cruz | Cat#sc-11048 |
| Anti-GPSM2 rabbit polyclonal | Abcam | Cat#ab84571 |
| Anti-NuMA rabbit polyclonal | Abcam | Cat#ab36999 |
| Anti-Laminin rabbit polyclonal | Abcam | Cat#ab11575 |
| Anti-β-actin mouse monoclonal | CST | #4970 |
| DAPI | Sigma | D9542 |
| Goat anti-Rabbit HRP | CST | #7074 |
| Goat anti-Mouse HRP | CST | #7076 |
| Biotin Anti-Mouse CD31(clone:390) | eBioscience | 13-0311-82 |
| Biotin Anti-Mouse CD45.2(clone:104) | eBioscience | 13-0454-82 |
| Biotin Anti-Mouse TER119(clone:TER-119) | eBioscience | 13-5921-85 |
| FITC Anti-Human/Mouse CD49f(clone:eBioGoH3) | eBioscience | 11-0495-82 |
| APC-CyTM7 Rat Anti-Mouse Ly-6A/E(D7) | BD Biosciences | 560654 |
| APC Anti-mouse CD326EpCAM (clone: G8.8) | eBioscience | 17-5791-82 |
| Anti-Mouse CD16/CD32(clone:93) | eBioscience | 14-0161-85 |
| Alexa Fluor^TM^ 488 donkey anti-mouse IgG(H+L) | Life Technologies | A21202 |
| Alexa Fluor 594 donkey anti-rabbit IgG(H+L) | Life Technologies | A21207 |
| Alexa Fluor 594 donkey anti-goat IgG(H+L) | Life Technologies | A11058 |
| Alexa Fluor 647 donkey anti-goat IgG(H+L) | Life Technologies | A21447 |
| ProLong^TM^ Gold antifade reagent with DAPI | Invitrogen | P36935 |
| IgG(H-270) rabbit polyclonal IgG | Santa Cruz | sc-66931 |
| IgG(3EB) mouse monoclonal IgG | Santa Cruz | sc-69786 |
