# Supplementary material for: E-cadherin bridges cell polarity and spindle orientation to ensure prostate epithelial integrity and prevent carcinogenesis in vivo
Source: PLoS Genet. 2018 Aug 17;14(8):e1007609. doi: 10.1371/journal.pgen.1007609 (PMC6115016; doi:10.1371/journal.pgen.1007609)
Supplement: S7 Table — (DOCX) [file pgen.1007609.s014.docx]

**S7 Table Primers used in this paper**

| Primers | | |
| --- | --- | --- |
| name | Primer1(5’ to 3’) | Primer2(3’ to 5’) |
| Probasin Cre | CTG AAG AAT GGG ACA GGC ATT G | CAT CAC TCG TTG CAT CGA CC |
| Geno-Cdh1 | GGG TCT CAC CGT AGT CCT CA | GAT CTT TGG GAG AGC AGT CG |
| Actin-QPCR | GGC TGT ATT CCC CTC CAT CG | CCA GTT GGT AAC AAT GCC ATG T |
| Cdh1-QPCR | AGG TTT TCG GGC ACC ACT TA | TGA TGC TGT CCC CAA GT |
| CK14-QPCR | GAC TTC CGG ACC AAG TTT GA | CTT GAG GCT CTC AAT CTG C |
| CK18-QPCR | ACT CCG CAA GGT GGT AGA TG | GCC TCG ATT TCT GTC TCC AG |
| Vim-QPCR | TCC ACA CGC ACC TAC AGT CT | CCG AGG ACC GGG TCA CAT A |
| Scrib #1 | ATAGAGCCTGCTCGCATTGAGGA | ctaCCGGTCGATGGAAGAGATGC |
| Scrib #2 | GATGATGCCCGGACCGGCGA | ctaCAGCAGGTTATTGGCCTGGTC |
| Scrib #3 | GTGGAGTCGGTGGACAAGC | ctaCTCCGTCTGGAACCGGAGCA |
| Scrib #4 | GAGCTGAGCCCTGAGGGCCCA | ctaGAGGTCTTCCACAGGGGTGG |
| Scrib #5 | GGCCCCCAGACCAGCACCT | CTAGGAGGGCACAGGGCCCA |
| Tgfbr2 | GACTGTCCACTTGCGACAAC | GGCAAACCGTCTCCAGAGTAA |
| Mki67 | ATCATTGACCGCTCCTTTAGGT | GCTCGCCTTGATGGTTCCT |
| Cxcl1 | ACTGCACCCAAACCGAAGTC | TGGGGACACCTTTTAGCATCTT |
| Tcirg1 | GAGACCTCAACGAATCCGTGA | CGATCCGTTTCCTCCTGGA |
| Cd74 | CGCGACCTCATCTCTAACCAT | ACAGGTTTGGCAGATTTCGGA |
| S100a11 | GCGGGAAGGATGGAAACAACA | TCATCATGCGGTCAAGGACAC |
| Osmr | GCATCCCGAAGCGAAGTCTT | GGGCTGGGACAGTCCATTCTA |
| Krt4 | TCGGCAGCAGAAGTCTTTACA | CAGCACCGTATCCTCCAACG |
| Cldn10 | GGCTACACATACAACGGACCC | TCCTTCTCCGCCTTGATACTT |
| Gjb1 | GCACGTAGCTCACCAACAG | TGATGACATAGGTCCACCACA |
| Gjb2 | CCCAGAAGGTCCGTATCGAAG | ACCAGACGTTGCATGAAGAAG |
| Cxcl16 | ACCCTTGTCTCTTGCGTTCTT | CAAAGTACCCTGCGGTATCTG |
| Mmp2 | ACCTGAACACTTTCTATGGCTG | CTTCCGCATGGTCTCGATG |
| Myo1f | CTCCGCAAACGCTTCATGG | CGCCCTGATAGAGGTCAATTTC |
| Arc | GGTAAGTGCCGAGCTGAGATG | CGACCTGTGCAACCCTTTC |
| Jak3 | ACACCTCTGATCCCTCAGC | GCGAATGATAAACAGGCAGGATG |
